# Engineering zonal cartilage through bioprinting collagen type II hydrogel constructs with biomimetic chondrocyte density gradient

**DOI:** 10.1186/s12891-016-1130-8

**Published:** 2016-07-20

**Authors:** Xiang Ren, Fuyou Wang, Cheng Chen, Xiaoyuan Gong, Li Yin, Liu Yang

**Affiliations:** Center for Joint Surgery, Southwest Hospital, the Third Military Medical University, Chongqing, 400038 People’s Republic of China; Orthopedic Department, 452nd Hospital Chinese PLA, Chengdu, 610021 Sichuan People’s Republic of China

**Keywords:** Chondrocyte, Collagen type II, Hydrogel, Cell density gradient, Total cell density, Biofabrication

## Abstract

**Background:**

Cartilage tissue engineering is a promising approach for repairing and regenerating cartilage tissue. To date, attempts have been made to construct zonal cartilage that mimics the cartilaginous matrix in different zones. However, little attention has been paid to the chondrocyte density gradient within the articular cartilage. We hypothesized that the chondrocyte density gradient plays an important role in forming the zonal distribution of extracellular matrix (ECM).

**Methods:**

In this study, collagen type II hydrogel/chondrocyte constructs were fabricated using a bioprinter. Three groups were created according to the total cell seeding density in collagen type II pre-gel: Group A, 2 × 10^7^ cells/mL; Group B, 1 × 10^7^ cells/mL; and Group C, 0.5 × 10^7^ cells/mL. Each group included two types of construct: one with a biomimetic chondrocyte density gradient and the other with a single cell density. The constructs were cultured in vitro and harvested at 0, 1, 2, and 3 weeks for cell viability testing, reverse-transcription quantitative PCR (RT-qPCR), biochemical assays, and histological analysis.

**Results:**

We found that total ECM production was positively correlated with the total cell density in the early culture stage, that the cell density gradient distribution resulted in a gradient distribution of ECM, and that the chondrocytes’ biosynthetic ability was affected by both the total cell density and the cell distribution pattern.

**Conclusions:**

Our results suggested that zonal engineered cartilage could be fabricated by bioprinting collagen type II hydrogel constructs with a biomimetic cell density gradient. Both the total cell density and the cell distribution pattern should be optimized to achieve synergistic biological effects.

## Background

Articular cartilage is a zonal tissue with a depth-dependent gradient of cells and extracellular matrix (ECM). From the articular surface to calcified cartilage, cartilage is usually divided into a superficial zone, a transitional zone, and a deep zone [1]. Although it is only a few millimeters in thickness, articular cartilage provides a nearly frictionless weight-bearing surface in diarthrodial joints and endures life-long extreme loading stress during limb motion. The functional properties of articular cartilage are correlated with its structural variations according to depth [2].

Articular cartilage is aneural, avascular, and alymphatic and has limited self-repair ability [3]. Once cartilage defects are caused by trauma, degenerative diseases, tumors, or other factors, they inevitably lead to progressive joint damage [4]. To date, it is still a substantial challenge for orthopedists to determine how to repair cartilage defects and restore joint function [5]. All treatments only relieve joint pain and delay the course of degeneration [6]. In the end, patients suffering from joint diseases have no choice but to undergo arthroplasty, which still has unresolved problems [7, 8].

Cartilage tissue engineering is a promising approach to repair and regenerate cartilage tissue through a combination of manufacturing technology and life science [9]. Aiming to mimic the articular cartilage in structure and function, the emphasis of recent research has shifted from homogeneous engineered cartilage to zonal engineered cartilage. Klein et al. [10] used chondrocyte subpopulations to produce zonal-specific ECM. Ng et al. [11] fabricated a chondrocyte-seeded agarose construct with distinct material properties in conjoined regions using a novel layered agarose technique. Woodfield et al. [12] achieved gradient distribution of cells and ECM by seeding chondrocytes into a polymer scaffold with a pore size gradient. However, little attention has been paid to the cell density gradient that is present in mammalian articular cartilage [13]. Zonal cartilage has a zonal chondrocyte density [14], different cell-cell communication [15], and different relationships between the cells and their environment [16–18].

In the present study, we hypothesized that the cell density gradient plays an important role in forming the zonal distribution of ECM. Zonal engineered cartilage was fabricated through bioprinting of collagen type II hydrogel constructs with a biomimetic chondrocyte density gradient. The constructs were cultured in vitro and harvested at 0, 1, 2, and 3 weeks for cell viability testing, reverse-transcription quantitative PCR (RT-qPCR), biochemical assays, and histological analysis. The objectives of the study were to offer a new strategy for producing zonal engineered cartilage and to assess the interactions between the total cell density and the cell distribution pattern to construct engineered cartilage.

## Methods

### Chondrocyte isolation and expansion

Chondrocytes were isolated from the knee joints of juvenile New Zealand white rabbits via enzymatic digestion. The animals were provided by the laboratory animal center and were sacrificed with the approval of the Animal Care and Use Committee at the Third Military Medical University. The cartilage was sequentially digested with 0.1 % trypsin in PBS at 37 °C and 5 % CO_2_ for 30 min, followed by treatment with 0.025 % collagenase II (Sigma, USA) in high-glucose Dulbecco’s modified Eagle’s medium (DMEM; HyClone, China) supplemented with 10 % fetal bovine serum (FBS; HyClone, China) and 1 % penicillin and streptomycin (P/S; Beyotime, China) at 37 °C and 5 % CO_2_ overnight. The isolated chondrocytes were expanded in 225 cm^2^ culture flasks in high-glucose DMEM supplemented with 10 % FBS.

### Collagen type II pre-gel preparation

Collagen type II was extracted from swine knees via pepsin treatment and salt precipitation, as previously described [19]. The swine knee joints were obtained from the nearby slaughter market and used with the approval of the suppliers. The collagen type II precipitant was dissolved in 0.15 M hydrochloride acid to obtain pre-gel at a 10 % (wt/vol) final concentration, which was then stored at 4 °C.

### Cell-laden construct fabrication

A bioprinter developed by our laboratory based on open-resource systems consists of robotic arms with three-axis motion, a stationary print platform, and a changeable print head. The print head on the X-axis robotic arm consists of piston-driven microscale depositional equipment. The bioprinter is controlled by software written by our laboratory.

Passage 3 chondrocytes were detached from culture flasks using 0.25 % trypsin (HyClone, China). Cell viability was assessed using the trypan blue exclusion test. The cells were counted with a hemocytometer and aliquoted according to different seeding densities. The collagen type II pre-gel was adjusted to pH 7.0 through a dropwise titration of 10 M sodium hydrate. The chondrocytes and collagen type II pre-gel were gently mixed in Eppendorf tubes at temperature below 20 °C.

**Fig. 1:**
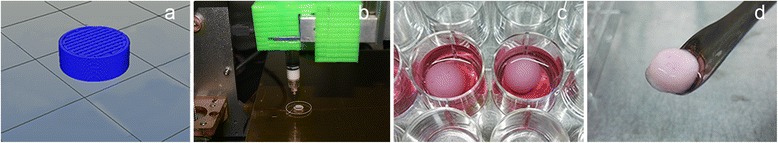
Images of the CAD model and the collagen type II hydrogel constructs. **a** Cylinder generated by CAD (4 mm radius and 6 mm height). **b** Printing on a sterile platform. **c** Constructs cultured in a 24-well plate. **d** Construct harvested at 3 weeks

**Fig. 2:**
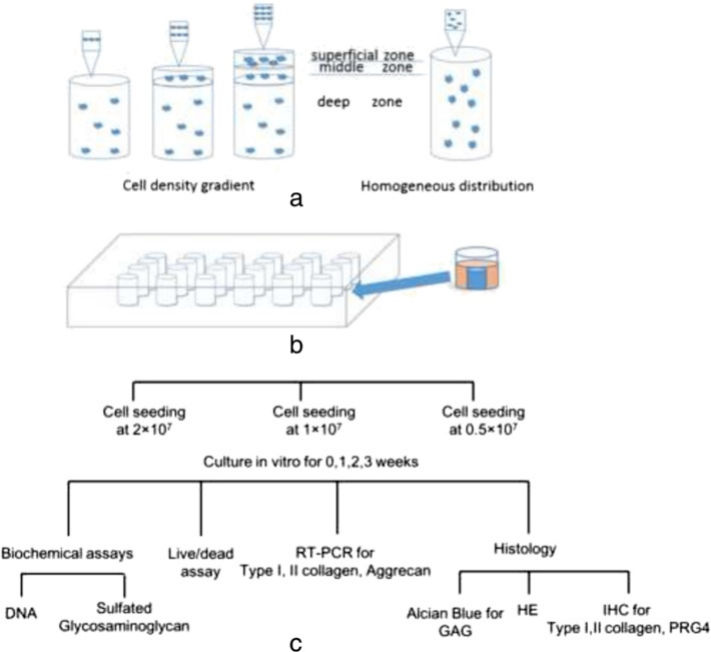
Study design schematic. **a** The construct with the cell density gradient and the construct with the homogeneous cell distribution were fabricated from bottom to top. **b** The constructs were cultured in a 24-well plate. **c** Three cell seeding density groups (2 × 10^7^, 1 × 10^7^, and 0.5 × 10^7^ cell/mL) were created. Constructs cultured in vitro were harvested at 0, 1, 2, and 3 weeks for biochemical assays, a live/dead assay, RT-qPCR and histological analysis

Our earlier study found that cell distribution patterns could not be well maintained in a printed hydrogel construct as thin as articular cartilage tissue due to zonal fusion caused by the hydrogel’s low mechanical strength. In the present study, a computer-aided design (CAD) model of a cylinder with a 6 mm height and a 4 mm radius was created, as shown in Fig. 1(a). The constructs were printed layer by layer, from the bottom to the top, on a sterile platform on a super-clean bench at a temperature below 20 °C, as shown in Fig. 1(b). A 25-gauge needle (0.25 mm inner nozzle diameter) was used. The deposition speed was set to 20 mm/s, and the layer thickness was set at 0.4 mm. Every construct had 15 layers. Three groups were created according to the total cell seeding density in the collagen type II pre-gel: Group A, 2 × 10^7^ cells/mL; Group B, 1 × 10^7^ cells/mL; and Group C, 0.5 × 10^7^ cells/mL. Based on Hunziker’s study [14], the total cell density within the articular cartilage of the human medial femoral condyle is 1 × 10^7^ cells/mL. Group B was designed to have a biomimetic total cell density, while Group A was designed to have a higher total cell density, and Group C was designed to have a lower total cell density. Each group included two types of construct: one with a cell density gradient and the other with a homogeneous cell distribution. According to previous research [14], the ratio of chondrocyte densities in the superficial zone (top 10 %) to the transitional zone (middle 10 %) to the deep zone (remaining 80 %) of normal adult human articular cartilage is approximately 3:2:1. As depicted in Fig. 2, the first 11 layers (deep 74 %) of the printed construct were designed to be the deep zone, the middle 2 layers (middle 13 %) were designed to be the middle zone, and the top 2 layers (top 13 %) were designed to be the superficial zone. The construct with the homogeneous cell distribution had a single cell density, which was equal to the total cell density. For the condition of the construct with the cell density gradient, the ratio of three zonal cell densities was 3:2:1, and the ratio of the three zonal volumes was 13 %:13 %:74 %. As the total cell densities were known for each group, the zonal cell densities shown in Table 1 could be obtained by solving a three-variable linear equation.

**Table 1:** Zonal cell density^a^ in each group

Group	Cell density gradient			Homogeneous distribution
	Superficial zone	Middle zone	Deep zone	
A	4.284 × 10^7^	2.856 × 10^7^	1.428 × 10^7^	2 × 10^7^
B	2.142 × 10^7^	1.428 × 10^7^	0.714 × 10^7^	1 × 10^7^
C	1.071 × 10^7^	0.714 × 10^7^	0.357 × 10^7^	0.5 × 10^7^

^a^Cell density unit: cells/mL

The fabricated constructs were cross-linked in a humidified incubator at 37 °C for 30 min [20] and were then moved into a 24-well plate and cultured in high-glucose DMEM supplemented with 10 % FBS, 1 % P/S, and 2.5 μg/mL amphotericin B (Beyotime, China) at 1.3 mL per well at 37 °C and 5 % CO_2_, as shown in Fig. 1(c). The culture medium was changed every other day. The constructs were harvested at 0, 1, 2 and 3 weeks, as shown in Fig. 1(d).

### Cell viability test

Cell viability was assessed using the Calcein AM (KeyGen Biotechnology Nanjing, China) and propidium iodide (PI, KeyGen Biotechnology Nanjing, China) molecular probes. Live cells were stained with Calcein AM (green), and dead cells were stained with PI (red). The constructs were sectioned and incubated in PBS containing 0.2 μM Calcein AM and 0.6 μM PI at room temperature for 1 h and were then rinsed in PBS. A full-thickness plane was imaged by fluorescent microscopy (Olympus IX71 microscope). The total cell number in each field of view could be quantified by separately counting green and red fluorescent dots. The percentage of live cells was calculated by dividing the number of live cells by the total cell number in 3 random images at 20× magnification.

### Gene expression analysis by RT-qPCR

Total RNA was extracted from the constructs using the Cartilage RNAout Kit (TIANDZ, China). The RNA concentrations and purity were measured using a NanoDrop 2000 (Thermo Scientific, USA). Reverse transcription was performed with the QuantiTect Reverse Transcription Kit (Qiagen, USA). Real-time PCR was performed with the QuantiFast SYBR Green Kit (Qiagen, USA). All procedures followed the manufacturers’ protocols. The expression levels of the collagen type I (COL1A1), collagen type II (COL2A1) and aggrecan (ACAN) genes were normalized to GAPDH, which was used as an endogenous control gene, according to the 2-△△CT method. The target gene primers were designed as follows: COL1A1 forward, 5-TTGGCAAAGAGGGCGGCAAAGGC-3, and reverse, 5-GAGCACCAGCAGGTCCGTCAGCA-3; COL2A1 forward, 5-ACGCTCAAGTCCCTCAACAACCAG-3, and reverse, 5-GTCTATCCAGTCACCGCTCTTCCA-3; ACAN forward, 5-ACTTCCGCTGGTCAGATGGA-3, and reverse, 5-TCTCGTGCCAGATCATCACC -3; and GAPDH forward, 5-GAAGGTGAAGGTCGGAGTC-3, and reverse, 5-GAAGATGGTGATGGATTTC-3.

### Biochemical analysis

After harvesting, the constructs were weighed and then digested for 24 h in 1 mL of 0.1 M PBS containing 2.5 U of papain (Beyotime, China), 5 mM cysteine-HCl and 5 mM Na2EDTA at 60 °C. The cell number was assessed based on the DNA content using Hoechst 33258 dye (KeyGen Biotechnology Nanjing, China), as previously described [21]. Hoechst 33258 is a fluorescent nucleic acid stain used to quantitate double-stranded DNA in solution. The fluorescence of the samples was converted to cell numbers using a cell-number standard curve created previously. The glycosaminoglycan (GAG) content was determined using the 1, 9-dimethylmethylene blue (DMMB; Jianglaibio, China) dye-binding assay and a shark cartilage chondroitin sulfate reference sample (C4348; Sigma, USA). The GAG content was normalized to the cell number to assess the single-cell biosynthetic activity.

### Histological analysis

The constructs were fixed in 4 % paraformaldehyde, dehydrated in a sequential ethanol series and embedded in paraffin. Sections (5 μm) were cut, deparaffinized and stained with hematoxylin-eosin (H&E) for cell distribution assessment and with Alcian blue for sulfated GAG assessment. For immunohistochemistry, rabbit polyclonal antibody Anti-Collagen I (bs-0578R; Bioss, China), Anti-Collagen II (bs-11929R; Bioss, China) and Anti-PRG4 (bs-1175R; Bioss, China) primary antibodies were used to detect collagen type I, collagen type II, and PRG4, respectively, synthesized by the rabbit chondrocytes. The primary antibodies were recognized using the Biotin-Streptavidin HRP Detection System (SP-9000; ZSGB-BIO, China) and a DAB kit (ZLI-9017; ZSGB-BIO, China). All procedures followed the manufacturers’ protocols.

### Statistical analysis

Statistical evaluation was performed using GraphPad Prism software (version 6.02). The data are presented as the mean ± standard deviation (SD) and were analyzed using analysis of variance (ANOVA) followed by Tukey’s honestly significant difference post hoc tests. Two-way ANOVA was used to reveal the differences in the total cell number, cell viability, GAG content and GAG/cell numbers among groups. One-way ANOVA was used to analyze the gene expression levels. In this study, *p* values less than 0.05 were considered statistically significant.

## Results

### Construct characterization

**Fig. 3:**
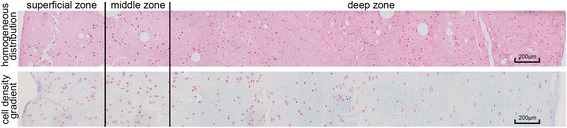
Representative images of H&E staining of the construct with the homogeneous cell distribution and the construct with the cell density gradient (scale bars, 200 μm)

The constructs were successfully fabricated and had uniform geometrical dimensions, with an average volume of 0.3 ± 0.05 mL. The physiochemical properties of the 10 % (wt/vol) collagen type II pre-gel adequately matched the requirements for hydrogel biofabrication [22]. As shown in Fig. 3, the results of the H&E staining showed that both the homogeneous and the gradient cell distribution patterns were effectively established and were maintained throughout the whole culture period.

### Cell viability

**Fig. 4:**
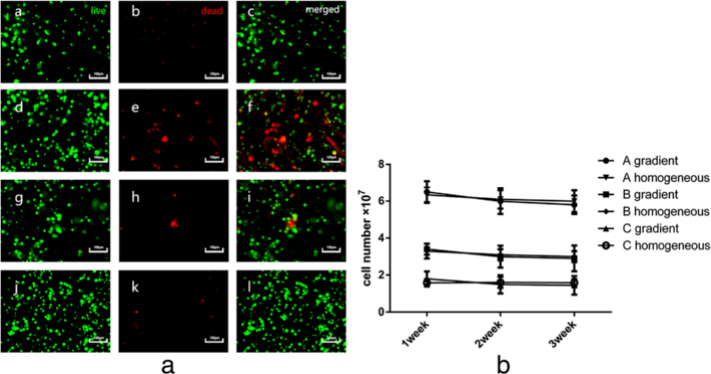
Cell viability after fabrication and the total cell number in the constructs. **a** The live cells were stained with Calcein AM (*green dots*), and the dead cells were stained with PI (red dots). The images in a, b, and c represent the construct with the homogeneous cell distribution in Group B on the first day after biofabrication; the images in d, e, and f represent the superficial zone of the construct with the cell density gradient in Group B after 1 week of in vitro culture; the images in g, h, and i represent the middle zone of the construct with the cell density gradient in Group B after 2 weeks of in vitro culture; and the images in j, k, and l represent the superficial zone of the construct with the cell density gradient in Group A after 3 weeks of in vitro culture (scale bars: 100 μm). **b** Total cell numbers in constructs in Groups A, B, and C after 3 weeks of in vitro culture

The trypan blue exclusion test showed that 98 ± 1 % of the chondrocytes that had detached from the culture flasks were alive. To assess the damaging effect of printing on cell viability, cell viability tests were performed on the first day after fabrication, as shown in Fig. 4(a). The average live cell percentage was 93 ± 3 %. No significant difference was observed between the two types of construct or among the different groups. As shown in Fig. 4(b), a slight decrease in the total cell number was observed in all groups during culture, but the difference was not statistically significant.

### Gene expression analysis

**Fig. 5:**
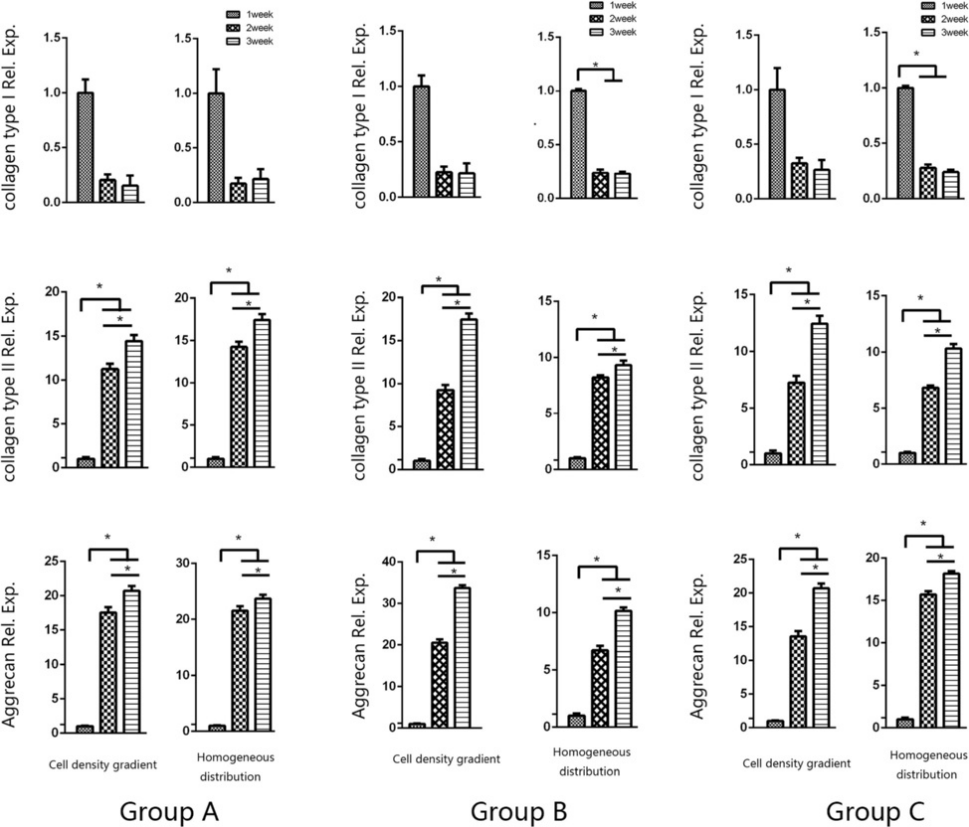
RT-qPCR analysis of the COL1A1, COL2A1, and ACAN expression levels in the construct with the cell density gradient and the construct with the homogeneous cell distribution in Groups A, B, and C after 3 weeks of in vitro culture. COL1A1: collagen type I; COL2A1: collagen type II; ACAN: aggrecan. The * indicates significance (*p* < 0.05). The error bars show the SD (*n* = 3)

To evaluate the phenotypic alterations of the chondrocytes in the collagen type II hydrogel constructs, the relative expression levels of COL1A1, COL2A1 and ACAN compared to GAPDH were determined by real-time PCR, as shown in Fig. 5. During the 3-week in vitro culture, COL1A1 expression remained low, and it was down-regulated throughout the culture period. However, the expression levels of both COL2A1 and ACAN were significantly increased (*p* < 0.05).

### GAG content quantification

**Fig. 6:**
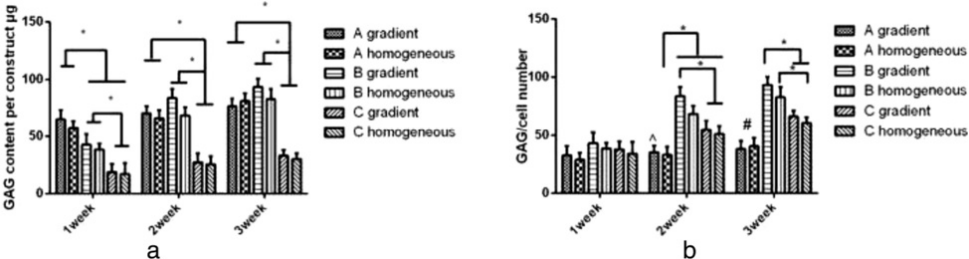
GAG content of the constructs and GAG content normalized to the cell number. **a** GAG content of the constructs in Groups A, B, and C after 3 weeks of in vitro culture. **b** GAG content normalized to the cell number. The * indicates significance (*p* < 0.05). The ^ indicates a significant difference between the gradient construct in Group A and the gradient construct in Group B, the homogeneous construct in Group B, and the gradient construct in Group C (*p* < 0.05). The # indicates a significant difference between Group A and Groups B and C (*p* < 0.05). The error bars show the SD (*n* = 3)

The total GAG contents in the constructs are shown in Fig. 6(a). During the first week of culture, the GAG content was positively correlated with the total cell density. Group A had the highest GAG content, and Group C had the lowest. During the second and third week, only Group C had a lower GAG content compared to Group A or B (*p* < 0.05). The differences between Group A and B were not significant.

To assess the average single-cell GAG production, the total GAG content was normalized to the cell number, as shown in Fig. 6(b). In the first week, no significant differences were observed among the three groups. In the last 2 weeks, the average single-cell GAG production in Group A was the lowest among the three groups. Because there was no significant decrease in the cell number, several of the cells in Group A might have entered the static state due to the limited nutrient supply. Group B had the highest average single-cell GAG production. Interestingly, there were significant differences between the construct with the cell density gradient in Group B and the constructs in Group A and C, although there was no significant difference within Group B. This result might be due to a synergistic effect of the total cell density and the cell distribution pattern. At 3 weeks, a significant difference was observed in the constructs with the homogeneous cell distribution between Group B and Group C, which might have been due to an effect of the total cell density.

### Alcian blue staining and immunohistochemical analysis of collagen types I and II and PRG4

**Fig. 7:**
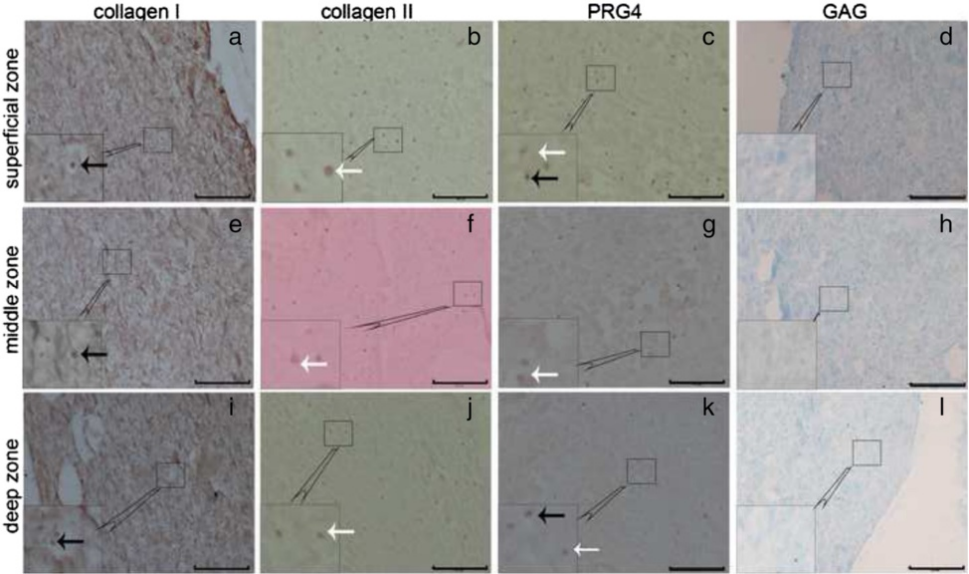
Zonal distribution of ECM in the constructs with the cell density gradient in Group A after 2 weeks of in vitro culture. The images in **a**, **b**, **c**, and **d** represent the superficial zone; the images in **e**, **f**, **g**, and **h** represent the middle zone; and the images in **i**, **j**, **k**, and **l** represent the deep zone. Images of collagen type I immunohistochemical (IHC) staining (**a**, **e**, and **i**); collagen type II IHC staining (**b**, **f**, and **j**); PRG4 IHC staining (**c**, **g**, and **k**) (scale bars: 100 μm). Images of Alcian blue staining (**d**, **h**, and **l**). The white arrows indicate positively stained cells, and the black arrows indicate negatively stained cells (scale bars: 200 μm)

To assess the ECM distribution in the constructs, Alcian blue staining for GAG and immunohistochemical analysis of collagen types I and II and PRG4 were performed. As shown in Fig. 7, the positively stained cells were brown, the negatively stained cells exhibited blue nuclei, and GAG in the matrix was also stained blue. Nearly all of the chondrocytes in the constructs stained positively for collagen type II, many stained positively for PRG4, and a few stained positively for collagen type I. In the constructs with the cell density gradient, the collagen type II, PRG4 and GAG contents were concentrated in the superficial zone and decreased with depth.

## Discussion

The ultimate goal of tissue engineering is to generate tissues or organs in vitro to repair and reconstruct impaired body function. As cartilage is aneural, avascular and alymphatic, it was predicted that cartilage would be one of the first tissues to be successfully replicated in the laboratory. However, this assumption proved to be incorrect. Engineered cartilage is fragile in vivo and has difficulties in integrating with the surrounding tissues [23]. One of the main defects of engineered cartilage is its lack of zonal properties compared to natural cartilage [24].

Chondrocytes are the sole resident cell type in articular cartilage [25]. A chondrocyte density gradient is one of the zonal properties of mammalian articular cartilage [13]. From the superficial zone to the deep zone in the human femoral condyle, the ratio of the zonal chondrocyte densities is approximately 3:2:1 [14]. In the present study, we printed collagen type II hydrogel constructs with a biomimetic chondrocyte density gradient and focused on the biological effects of the cell density gradient and the total cell density. To compare the two different cell distribution patterns at a specific total cell density, two types of construct were designed: one with a cell density gradient and the other with a homogeneous cell distribution. To evaluate the interactive effects of the total cell density and the cell distribution patterns, three groups were created according to the total cell seeding density. The results of the study demonstrated that total ECM production was positively correlated with the total cell density in the early culture stage, that both the cell distribution and the total cell density had effects on the cells’ biosynthetic ability, and that the cell density gradient distribution resulted in a gradient ECM distribution.

A primary goal of biofabrication is to achieve high cell viability in constructs [26]. When cells are deposited within biomaterials using a bioprinter, they are exposed to shear stress, and damage may occur [27]. Attention should be paid to both the rheological parameters (e.g., viscosity) of the printing material and the printing parameters (e.g., dispensing pressure and nozzle size) [28, 29]. In our study, all chondrocytes were encapsulated in a 10 % (wt/vol) collagen type II pre-gel. Thus, the viscosity of the mixture of chondrocytes and pre-gel depended on the cell density. With the same printing parameters, the superficial zone of the construct with the cell density gradient in Group A had the highest risk of cell damage because it had the highest cell density. Nevertheless, the cell viability test showed that 93 ± 3 % of the chondrocytes were alive after printing. No significant difference in cell viability was observed between the two types of constructs or among the three groups, which was consistent with previous studies [22]. The cell viability was also determined by quantifying the total cell number. As shown in Fig. 4(b), there were no significant changes in the total cell numbers in any construct throughout the culture period. Cell apoptosis might account for the slight decrease.

As a basic ingredient in tissue engineering, scaffolds should provide a platform that directs cell adhesion, proliferation, and ECM synthesis. Designing a biomimetic scaffold to accommodate cells in a suitable milieu is one of the most important strategies in tissue engineering [30]. Hydrogels possess many characteristics of a natural ECM and are widely used for three-dimensional cell culture [31, 32]. In articular cartilage, chondrocytes dwell in the natural collagen type II hydrogel networks, the interstitial voids of which are filled with a large amount of fluid components and some GAG [1]. Based on the biomimetic component idea, we chose collagen type II hydrogel to construct engineered cartilage. The results of the RT-qPCR and immunohistochemical analyses of collagen types I and II showed that chondrocytes cultured in collagen type II hydrogel retained their chondrogenic phenotype. Meanwhile, many studies have demonstrated that collagen type II has the ability to retain the biological functions of chondrocytes and is appropriate for engineering cartilage [33–35].

Cells have a core role in tissue engineering systems. The main function of cells is to synthesize and maintain ECM. Once cells settle down on scaffolds, they sense the environment and react accordingly. Both the cell seeding density and the cell distribution pattern are important parameters when designing a tissue engineering construct. It has been demonstrated that the cell seeding density is a positively correlated with ECM production [36, 37] and that an anisotropic distribution of cells leads to an anisotropic distribution of ECM [12]. However, few studies have investigated these two parameters simultaneously and quantitatively. In the present study, we evaluated the two factors in one system by comparing two cell distribution patterns at three total cell densities. The results showed that the constructs in Group B (1 × 10^7^ cells/mL total cell density), and particularly the construct with the cell density gradient had two advantages, namely, improved total GAG production and single-cell biosynthetic ability, over the constructs in the other two groups at 2 weeks of in vitro culture. The results showed the synergistic effects of the cell distribution pattern and the total cell density. Meanwhile, the constructs with the cell density gradient had the advantage of zonal ECM properties similar to those of natural cartilage compared to the constructs with the homogeneous cell distribution.

In previous studies [12], a cell gradient distribution was achieved using a scaffold with a pore size gradient, in which the cells were randomly settled on the surface of the polymer fibers. The zonal cell density and the total cell number in the scaffold could not be quantified. Furthermore, the cell distribution pattern in the scaffold was not stable during culture. In addition, different pore sizes may have different biological effects on cells [38]. In our study, chondrocytes and collagen type II pre-gel were mixed to produce the required cell density. The zonal proportions and cell density gradient in articular cartilage were well duplicated in the construct by bioprinting. The cell distribution patterns remained stable throughout the culture period. The results of the immunochemistry and Alcian blue staining demonstrated that the new ECM had a gradient distribution in the constructs with the cell density gradient. This observation was more meaningful for PRG4, which is a specific protein of the superficial zone that is responsible for surface lubrication [24, 39].

Our study provided a new strategy to construct zonal engineered cartilage. Compared to traditional scaffold fabrication methods, hydrogel bioprinting has the advantages of being able to fabricate constructs with an arbitrary geometry on the macro-scale, particularly for constructs with a curved surface, and to accurately control the cell distribution in constructs on the micro-scale. Zonal engineered cartilage was fabricated by bioprinting a construct with a cell density gradient. This technique is easy to perform, and there are no difficulties in isolating and expanding the chondrocyte subpopulations. More importantly, by optimizing the total cell density and the cell distribution pattern, the chondrocytes can be induced to exhibit maximum biosynthetic ability. Researchers may no longer need to wait for long periods to allow chondrocytes to expand to achieve maximum ECM deposition in a scaffold. In addition, the study showed that structurally biomimetic engineered cartilage could be constructed by hydrogel biofabrication. The constructs with a cell density gradient could be a good platform for investigating cell-cell communication and the relationships between cells and their environments.

The study also has certain limitations. First, articular cartilage is a weight-bearing tissue. Mechanical stimuli play an important role in mediating chondrocytes’ biological functions [18]. Zonal chondrocytes sense the zonal mechanical environment before producing a specific ECM. In this study, however, the results were only observed in static culture. Further studies should be performed to observe more differences between the two types of constructs in an appropriate mechanical environment (e.g., via culture in a bioreactor). Second, constructs with a biomimetic cell density gradient should be evaluated in vivo to observe whether these constructs are able to better integrate with the surrounding tissues and produce a satisfactory clinical outcome. Furthermore, we did not pay much attention to the effects of the total cell density and the cell distribution pattern on the subpopulation phenotype. In future studies, effort should be devoted to retaining and/or creating a subpopulation phenotype [40]. It would also make sense to fabricate engineered cartilage with a biomimetic distribution of zonal marker proteins.

## Conclusions

In this study, we printed collagen type II hydrogel constructs with a biomimetic chondrocyte density gradient and focused on the biological effects of the cell distribution and the total cell density. The results from the in vitro static culture showed that total ECM production was positively correlated with the total cell density in the early culture stage, that the cell density gradient distribution resulted in a gradient distribution of ECM, and that the chondrocytes’ biosynthetic ability was affected by both the total cell density and the cell distribution pattern. The results suggested that zonal engineered cartilage could be constructed by bioprinting a collagen type II hydrogel construct with a biomimetic cell density gradient. Both the total cell density and the cell distribution pattern should be optimized to obtain synergistic biological effects.

## Abbreviations

ACAN, aggrecan; ANOVA, analysis of variance; CAD, computer-aided design; COL1A1, collagen type I; COL2A1, collagen type II; DMEM, Dulbecco’s modified Eagle’s medium; ECM, extracellular matrix; FBS, fetal bovine serum; GAG, glycosaminoglycan; H&E, hematoxylin-eosin; P/S, penicillin and streptomycin; PI, propidium iodide; SD, standard deviation
